# Carcinoma Erysipeloides With Clinical and Dermatoscopic Features: An Overlooked Clinical Manifestation of Breast Cancer

**DOI:** 10.7759/cureus.23445

**Published:** 2022-03-24

**Authors:** Francin Alexis, Luke R Leggett, Nikita Agarwal, Zachary Bakhtin, Banu Farabi

**Affiliations:** 1 Internal Medicine, St. Peter's University Hospital, New Brunswick, USA

**Keywords:** breast carcinoma skin metastasis, inflammatory breast cancer, dermato-oncology, and cutaneous breast carcinoma, metastases, breast cancer, carcinoma erysipeloides

## Abstract

Carcinoma erysipeloides (CE) is an atypical finding among women with breast cancer. CE clinically presents as an erythematous patch or plaque resembling superficial skin infections such as cellulitis or erysipelas. CE can also be the first indication of an underlying breast cancer. Therefore, it is imperative for clinicians to recognize this rare entity for early diagnosis and improving prognosis and outcomes in breast cancer patients. Here, we present a 68-year-old female patient with a history of breast cancer, who developed recurrence of cancer with typical clinical features of CE. Hence, we aim to increase awareness of this rare entity. We also report the dermatoscopic features of CE, which to the authors’ knowledge have not been previously documented in literature.

## Introduction

While skin metastases can occur in other forms of malignancy, the incidence is found to be the greatest in breast cancer. Clinically it can present as a unilateral erythematous lesion typically seen on the anterior chest wall and can spread to other regions of the body such as the back, proximal arm, and can even cross the midline of the body [1]. Cutaneous metastasis from breast cancer shows various clinical manifestations including telangiectasia, papules/plaques or indurated nodules, “en cuirasse” carcinomas, alopecia neoplastica, and zosteriform eruptions. The most common presentation is skin papules or nodules which have been reported in more than 80% of the reported cases [2,3]. Carcinoma erysipeloides (CE) can present with features that resemble erysipelas, a skin infection caused by Group-A Streptococci (GAS), as well as cellulitis. Thus, appropriate treatment options may be delayed due to improper identification of cutaneous cancer manifestations. Any delay in treatment may be detrimental to the patient, which is why immediate recognition and consideration of treatment should be established to mitigate the systemic spread of the disease. Herein we present a case of CE in a patient with previously diagnosed breast cancer which heralded a recurrence of the disease.

## Case presentation

**Figure 1:**
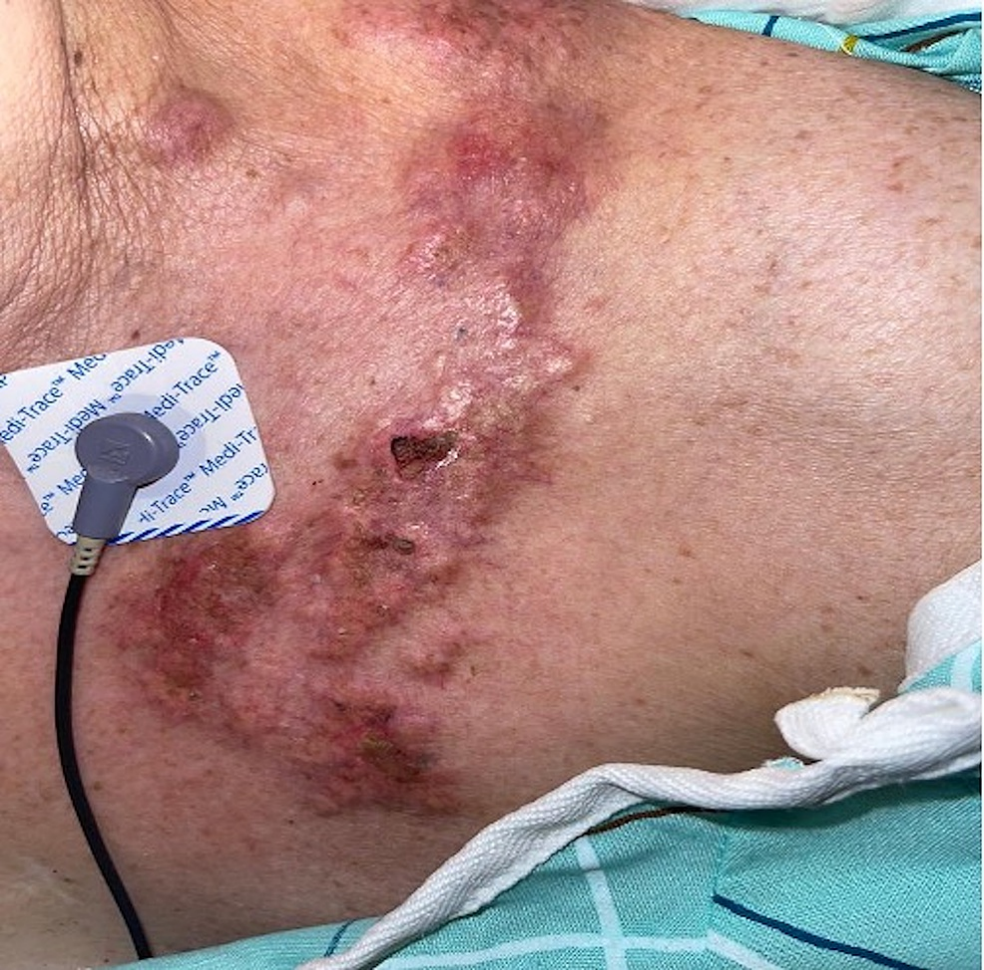
Superomedial left chest image Clinical image of carcinoma erysipelodes showing an indurated linear erythematous plaque lesion on the superomedial left chest

A 68-year-old female with a past medical history of ER-positive, HER2-neu negative breast cancer in remission was admitted to the hospital with black-colored stools and diarrhea. The patient had been diagnosed with pulmonary embolism eight days prior and started on apixaban 5 mg BID (twice in a day) treatment. Shortly after starting apixaban, the patient began to have increased bowel movements with black stools and presented to the emergency room for suspicion of upper gastrointestinal bleeding. Upon physical examination, there was a notable erythematous, nodular, 10 x 15 cm fixed lesion on the left upper medial chest (Figure 1).

**Figure 2:**
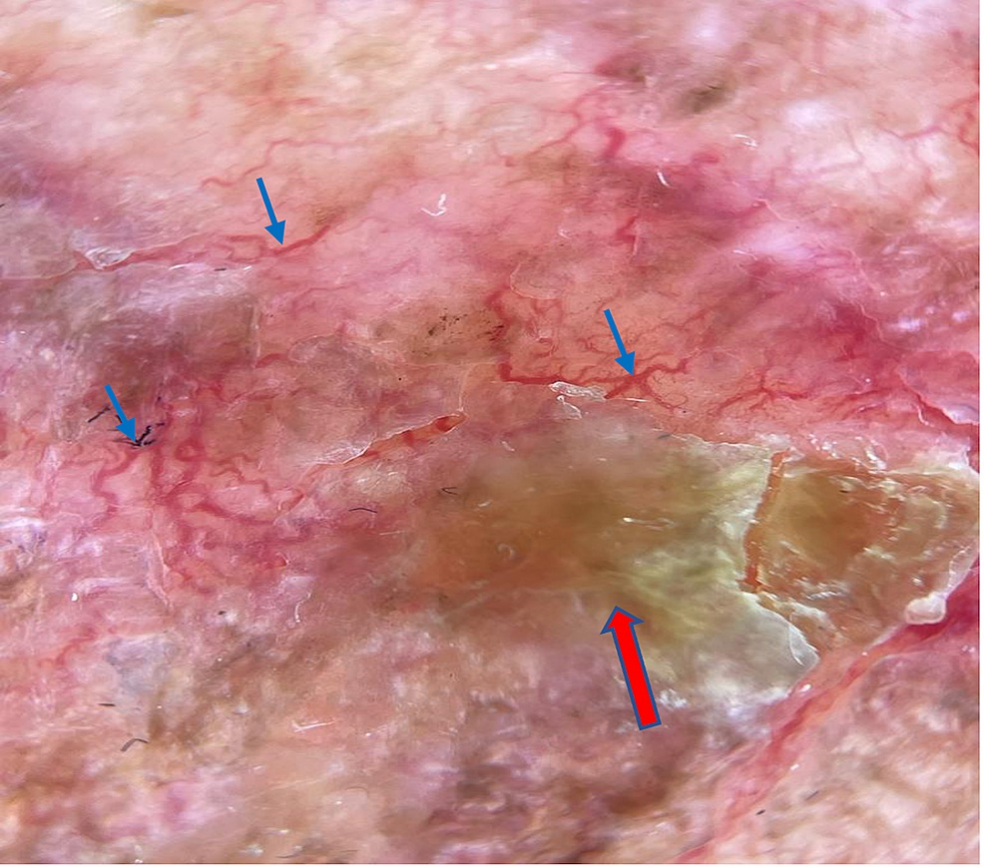
Dermatoscopic examination image The carcinoma erysipeloides image is showing an erythematous-orange background with telangiectasia (blue arrows), yellow crusting and dyspigmentation (red arrow)

The dermatoscopic examination revealed a nodular heterogeneous lesion with dyspigmentation and multiple telangiectatic vessels (Figure 2). A skin biopsy of the skin lesion was performed and demonstrated clusters of tumor cells concentrated within the dermal layer. These findings were consistent with cutaneous metastasis secondary to breast cancer. The patient has been diagnosed with CE. The hematology department was consulted for recurrence of the breast cancer, and the patient was scheduled for outpatient radiotherapy.

## Discussion

In many diseases, the skin only reveals a small scope of what is happening within the deeper structures. People, in particular women, with breast cancer who present with unexplained skin eruptions, should be further evaluated. Breast cancer is the second most common cause of cancer in women and number one in cancer-related deaths. Several studies have mentioned the prevalence of breast cancer, with approximately 268,000 people being diagnosed with breast cancer annually in the US [4]. Of the patients diagnosed with breast cancer, it is reported that up to 32.7% will develop metastases. With infiltrating ductal carcinoma comprising 22.7% and lobular carcinoma making up the other 4.7% [5]. Cutaneous metastases in adult females are most commonly a direct result of primary breast cancer [6]. CE, as the first manifestation of breast cancer, is very rare and accounts for about 2%-5% of all cases [7,8]. As a result, CE can serve as a screening tool for reoccurrence.

CE can present with different cutaneous manifestations. This can consist of irregular, ill-defined plaques or patches which may be either erythematous or indurated in nature. The other distinctive clinical patterns of metastasis almost exclusive to breast cancer include telangiectatic carcinoma and carcinoma en cuirasse. Carcinoma telangiectoides is manifested by red papules and telangiectasias, and carcinoma en cuirasse is manifested by dusky, translucent skin with an orange peel appearance which mimics morphea due to the associated induration [1]. It can also be confused with radiation dermatitis in certain patients [6]. CE may go undiagnosed for an extended period of time. Therefore, those with repeated failed treatments and a history of breast cancer should prompt physicians to further investigate in obtaining a dermatoscopic examination and skin biopsy for confirmation. The biopsied lesion will demonstrate the cancer cells dependent on the individual's breast cancer type. The dermatoscopic features of CE have never been reported in the literature previously. In parallel with the clinical picture in our patient, we observed an erythematous telangiectatic lesion with dyspigmentation under polarized dermatoscopy with an orange hue. This could reflect the packed tumor cells infiltrating the papillary dermis in our case. Further studies regarding dermatoscopic features of CE should be conducted to conclude this relationship.

Taken altogether, it is important that clinicians rule out CE in patients presenting with skin lesions, especially in those with an active or previous history of breast cancer. The lesion itself can serve as a tumor marker underlying the progression or recurrence of advanced carcinoma of the breast [5].

## Conclusions

Clinicians should be aware of CE in patients presenting with cutaneous lesions. Albeit rare, it may be the first sign of breast cancer recurrence, allowing for early intervention to take place. In addition, those with a history of breast cancer should be made aware of possible skin manifestations so they can promptly seek care at the first sign of any suspicious skin changes.
